# Positive Development Based on the Teaching of Personal and Social Responsibility: An Intervention Program With Institutionalized Youngsters

**DOI:** 10.3389/fpsyg.2022.792224

**Published:** 2022-03-24

**Authors:** Paulo Martins, António-José Gonzalez, Margarida Pedroso de Lima, João Faleiro, Luís Preto

**Affiliations:** ^1^Sport Psychology Laboratory, Faculty of Human Kinetics, University of Lisbon, Lisbon, Portugal; ^2^ISPA – University Institute of Psychological, Social and Life Sciences, Lisbon, Portugal; ^3^Department of Clinical Psychology, Faculty of Psychology and Educational Sciences, University of Coimbra, Coimbra, Portugal; ^4^Faculty of Human Kinetics, University of Lisbon, Lisbon, Portugal

**Keywords:** life skills, personal responsibility, social responsibility, sport, football

## Abstract

From the standpoint of the school settings, sport participation constitutes a key strategy concerning the manifestation of positive behaviors that result from the development of personal and social responsibility. Based on the TPSR model, the goal of this study was to evaluate the effects of an intervention geared toward teaching life skills through sport to youngsters who had been committed. The participants were evaluated before and after the intervention. After the initial evaluation, they were randomly assigned to the experimental and control groups. The experimental group took part in the community football program, while the control group attended physical education classes not based on the TPSR model. The experimental model consisted of 3 weekly sessions over the course of 6 weeks, which totaled 18 sessions. This investigation supplied empirical evidence concerning the potential of community sport programs in the teaching and development of life skills deemed necessary for an adequate reintegration of such at-risk youngsters. The model was shown to be valid both in stimulating changes of attitudes and in promoting the adherence to socially positive behaviors. The effectiveness of the model, as well as its unique approach, make its application attractive to both the youngsters and the professionals. This program facilitates the training of youngsters to act, in the sense of promoting both their autonomy and the acquisition of a system of ethics and moral values within a culture of responsibility for how decisions affect the individual and the community. Finally, this intervention generated empirical support in favor of the argument that sport constitutes a unique opportunity within the educational process to establish values, beliefs, attitudes, and practical habits pertaining relationships and cooperation that generate social responsibility in individuals.

## Introduction

Positive psychology emphasizes the importance of developing life skills, which allows individuals to face life’s challenges with hope and gratitude (Peterson and Seligman, 2004; Lopez and Snyder, 2009; Arslan and Wong, 2022). The development of life skills implies the organization and supervision of practices geared toward the acquisition of moral codes and values (Lee and Whitehead, 1999). There is, within the sport literature, an extensive body of publications focused on programs for positive youth development (PYD) (Gould and Carson, 2008; Camiré et al., 2011, 2018; Kendellen and Camiré, 2019; Strachan et al., 2021). There are also several publications on the pedagogical models that have been used successfully for 40 years to foster PYD through physical education (PE) and sport, thus implementing the teaching of personal and social responsibility model (TPSR model; Hellison, 1985, 2011). Specific accounts of the latter include the successful use of the TPSR model in the Portuguese pre-school setting (Santos et al., 2020); the hybrid combination of the TPSR model and the Teaching Games for Understanding approach in the context of PE (García-Castejón et al., 2021); and the use of the TPSR model in competitive youth sport (Carreres-Ponsoda et al., 2021). Case in point, social and emotional learning have been identified at the pre-school level (Santos et al., 2020), while the enhancement of personal and social responsibility, prosocial behavior, and self-efficacy were reported in competitive youth sport (Carreres-Ponsoda et al., 2021). Furthermore, the combined use of the TPSR model and the Teaching Games for Understanding approach showcased a heightened intention to be physically active, as well as the improvement of the psychological variables motivation, responsibility, and enjoyment in PE participation (García-Castejón et al., 2021). In sum, through sport, life skills can be trained in a variety of ways and applied in a variety of contexts, which will enable positive youth development (Brunelle et al., 2007; Martinek and Hellison, 2016). Sport can generate the positive development of young people. As it is organized, it requires commitment over time and includes interpersonal relationships (Geldhof et al., 2013). Among the several effects of sport that have been studied thus far, the particular topic of personal and social responsibility has been gaining greater relevance (Martins et al., 2017). Therefore, from an educational point of view, sport is an essential strategy in promoting personal and social responsibility, resulting in positive social behavior among young people (Hellison and Walsh, 2002; Hellison and Martineck, 2006). Personal and social responsibility (PSR) is a form of positive development that will provide a successful transition to adulthood (Hellison and Martineck, 2006; Escartí et al., 2010). The fundamentals of the model “Teaching Personal and Social Responsibility” (TPSR) are made distinct by two factors, personal responsibility and social responsibility (Hellison, 2011; Martins et al., 2017). A definition vastly accepted within the literature presents personal responsibility as the act of taking responsibility for one’s own life, as well as for the behaviors that result from the choices made. Personal responsibility generates the implication that certain tasks are mandatory for the individual, given that the latter is a part of society. On the other hand, social responsibility can be conceptualized as the degree of connection between the individual and the other members of the community, thus highlighting the level of concern regarding helping and supporting others (Hellison, 2011; Martins et al., 2015).

The TPSR model was developed by having in mind a coherent and well-thought-out and integrated development of the individual. This was based on a setting of self-regulation (personal responsibility), where inter-relationships are centered on caring for others (social responsibility; Hellison, 2011). This model looks to fulfil five goals: (a) self-regulation, (b) self-motivation, (c) self-direction, (d) concern for others, (e) transfer to “outside of the gym” (Hellison, 2011). The contexts in which they were applied include three distinct but complementary settings: 1—the school context; 2—extracurricular activities and/or community programs in low income neighborhoods, and 3—professional training. For example, in New Zealand skills, such as self-regulation, concern for others, and active participation in an organized and contributive manner are the key points of the curriculum of PE (Gordon et al., 2011). The academic setting within the Portuguese context includes subjects meant to supply students with skills mainly geared toward the professional setting. For example, Carbonero et al. (2015) conducted a study with 235 students meant to evaluate the attitudes pertaining to personal and social responsibility among teenage youngsters. The results of this study support the thesis that, regardless of one’s academic achievement, there is an improvement of the variables related to personal and social responsibility, as well as of one’s general attitudes (satisfaction and effort), among the students pertaining to academic activities. The study also highlights that greater levels of PSR are associated with greater performance levels in the academic setting (Carbonero et al., 2015). In another study in the United States, Wright et al. (2010) also encountered statistically significant changes within the variables attendance and punctuality of the students during the period of implementation of the TPSR model in a class of Tai-Chi.

In fact, the relevance of the TPSR model is such that several countries, despite having an already heavy school curriculum, call for sport-based extracurricular activities. The TPSR model can be, within this approach, a plausible alternative in the school curriculum, bringing about outcomes of positive development in youngsters (Catalano et al., 2004; Martins et al., 2015; Arslan and Wong, 2022).

Youngsters at risk, or displaying behaviors of risk, constitute a group that usually displays behaviors that fail to match the expectations of the role, thus resulting quite often in harmful situations both to oneself and to the community (Damon, 2004). In fact, most of these youngsters live in low income neighborhoods, in which the prevalence of drug use, violence, theft, gang association, and skipping school act as facilitating conditions of risk behaviors (Damon, 2004). Interventions inspired by the TPSR model have been developed in the United States in summer camps and extracurricular activities (Watson et al., 2003). For example, in the United States Riley and Anderson-Butcher (2012) tested the application of the program in summer camps (in which 600 youngsters took part), and found a positive impact, not only among the youngsters, but also among their parents, family and the community at large. Furthermore, the study supported the notion that acquisitions do transfer to one’s life outside of the program, thus representing life skills. Concerning these strategies, the authors also highlight that the intervention ought to be conducted in a progressive manner, while valuing the qualities of each individual and making them capable of inter-relationships. Ultimately, when applying strategies based on the TPSR model, youngsters gain conscience of their own actions and, upon doing so, increase their self-control. This brings about an alignment of behaviors, meaning that their actions are associated with positive behaviors (Riley et al., 2017).

Other TPSR inspired programs that have been developed in the United States include the Chicago-based Beyond the Ball program, led by Amy and Rob Castañeda; the Get Ready program, managed by John McCarthy at Boston University; the Illinois-based Project Leadership, run by Paul Wright and Jenn Jacobs; and the Youth Leader Corps program, active at the University of North Carolina under Tom Martinek (Martinek and Hellison, 2016).

The TPSR model has, additionally, also been made to integrate university-level professional programs. Such cases include a program at the university of Regina (Canada), run by Nick Frosberg; a teacher education program at San Francisco State University, run by Dave Walsh; a program at the Adelphi University, run by Meredith Whitley; and a master’s degree in community youth sport development, run by the aforementioned Tom Martinek at the University of North Carolina (Martinek and Hellison, 2016).

In Portugal, sport has been assuming an important role in facilitating approaches geared toward the generation of psychological wellbeing, moral and emotional development of youngsters, and interpersonal skills (Gouveia et al., 2003; Martins et al., 2015, 2017). Therefore, physical activities are considered an optimal environment in which many positive qualities and attributes are developed, namely, the positive development of youngsters (Wright and Li, 2009). In short, when sport is approached from an educational perspective, it is considered an excellent vehicle for developing positive social behaviors, thus playing a fundamental role in both the upbringing and the behavior of youngsters (Hellison and Walsh, 2002; Hellison and Martineck, 2006).

In this context, the Commission of Support and Control for Portuguese Educational Centres considers that, of the 186 youngsters under court measures, 144 were identified as potential participants of the educational-based program. The mission of an educational center is to allow for youngsters under educational supervision to acquire knowledge, skills, and social values that target their successful reintegration, both socially and professionally (DGRSP, 2019). The purpose is to embrace youngsters by following up the court-of-law implemented measures, as established by the law in effect. The latter includes educational measures, scenarios of foster-parenting, being committed for an evaluation of personality, and the order of detention (DGRSP, 2019). The Educational Intervention Project is a fundamental instrument that structures and organizes the interventions within the educational centers. Even today, the judicial measures applied to young offenders are merely punitive and without any objective aimed at teaching life skills enabling their re-insertion into the community. The goal is that the youngster is able to both understand and internalize the fundamental values, rules and socio-legal norms. The latter ensure social interactions and favor the development of the person and citizen within the scope of responsibility (DGRSP, 2019). As stated earlier, though several variables may be taken into account in order to generate personal and social education, sport constitutes, as an organized form of physical practice, a highly significant opportunity for the education and positive development of youngsters (Hellison, 1973, 2011; Martins et al., 2015). Physical activities in general, and sport in particular, include a high potential for significantly impacting the personal development of youngsters, thus contributing to their development as people. Therefore, sport training and competition, namely, for youngsters, should also take on an educational dimension, which ought to play-out in the form of projects that target personal and social development (Martinek and Hellison, 2016).

The goal of this study is to evaluate both the implementation and the effects of an intervention based on the model for personal and social development through sport. It is our understanding that the outcome of this study may contribute toward an improved theoretical and practical understanding of the development of youngsters. As such, a subsequent exploratory approach will facilitate the study of a model that best fits the specific environment that characterizes the educational centers managed by the government. This approach, never before attempted with institutionalized youngsters at risk, will shed light on the impact that an approach based on sport and organized physical activity has on their modification.

## Materials and Methods

### Outline

The experimental model lasted 6 weeks, for a total of 24 sessions, more specifically three weekly sessions of an hour and a half each (for a total of 18 sessions) of sport and physical practice through football, and six sessions of an hour and a half in a classroom. These sessions were devoted to giving meaning to the development of the concepts that make up the TPSR model through a cognitive and affective process. The latter included a particular focus being placed on the creation of behavioral change (Escartí et al., 2013).

During the initial instruction of each training session, one of the several themes within the program was explored. This procedure looks to establish an understanding of the levels and goals of the TPSR model. The set of 18 training sessions included the development of 1 theme per session (Table 1).

**Table 1:** Levels of responsibility, core behaviors, sessions, and format lessons during the intervention.

Responsibility levels	Responsibility behaviors to be achieved	
1. Respect for the rights and feelings of others	Self-controlling impulses. Resolving conflicts peacefully.	
2. Participation and effort	Participating in all activities. Persisting in difficult tasks.	
3. Self-direction	Working independently. Setting and working toward goals autonomously. Making good choices.	
4. Leadership	Helping others. Leading others. Considering other youngster’s interests and needs.	
5. Transfer	Understanding and applying these skills outside of the gym setting	
**Lessons format**		
**Activity**	**Content**	**Temporal elements**
Relational time—helps to create a welcoming environment and establish personal relationships with students	Informal one on one interactions teacher–students. Ask students how their day is going, and discuss things that may be coming up or going on in their lives. Obs. Can happen whenever an opportunity arises, (i.e., before or after the lesson, or even during the lesson while the students are in transitions).	Before lesson
Awareness talk—brief structured meeting that officially begins the lesson. The teacher can go over the plan for the day, invite input from students, and most importantly, remind them of the goals and objectives of the program.	Depending on the stage of the program, a teacher might be focusing on a particular aspect of responsibility or just reminding students of the overall emphasis in the program, (e.g., how they conduct themselves and how they treat others).	Usually just a few minutes (0–5′)
Sport activity lesson time—involve appropriate physical activity, exercise or sport content.	The teaching strategies employed during this activity time shift power to the youngsters and put them in responsible roles. The key is to integrate the teaching of responsibility with the teaching of the specific physical activity.	Constitutes the majority of the lesson (20′-30′)
Group meeting—After most of the physical activity is completed, the teacher gathers students to discuss the lesson.	This provides an opportunity to share the youngsters’ opinions about the lesson, make suggestions, and comment on the group’s performance and cohesion. If the day’s activities had involved student leadership, this Group Meeting could provide a safe and structured opportunity for students to provide feedback to their peer leaders and vice versa.	Only a few minutes are necessary for this meeting (3′-5′).
Reflection time—Students are asked to reflect on their own attitudes and behaviors during the lesson.	Using the responsibility levels as reference points, the teacher prompts the youngsters to think about their performance that day relative to each other. Depending on the number of students and the climate of the program, the teacher may have students share their self-reflections at this time verbally, with a hand signal (thumb up for “great,” sideways for “okay,” or down for “needs work”), or writing in a reflective journal.	For the last few minutes of the lesson.
**Core Theme’s by Lesson**		
Lesson 1 Lesson 2 Lesson 3 Lesson 4 Lesson 5 Lesson 6 Lesson 7 Lesson 8 Lesson 9 Lesson 10 Lesson 11 Lesson 12 Lesson 13 Lesson 14 Lesson 15 Lesson 16 Lesson 17 Lesson 18	Dedication Confidence Enthusiasm Cooperation Affectivity Honesty Tolerance Fair play Camaraderie Overcoming of self Transfer to one’s life Responsible participation Nothing to fear Everyone has a function Sportive spirit *VS* Values Self-motivation Concern with others Collective goals *VS* Personal goals	

*Retrieved from* *https://www.tpsr-alliance.org/tpsr-model/responsibility-levels*.

The themes were chosen according to the practical exercises used in each football training session, so as to establish an inter-relationship between cognition (knowledge of the game and implicit rules) and behavior (technique and tactics, behaviors related to fair play). For example, here are the topics for session 11:

Theme: Transfer to life; formal game of 5 against 5. In this session it is sought that the youngsters comply with levels 4 and 5 of the TPSR model, “Caring” and “Transference for outside of the game,” respectively (assuming that the path over the course of the sessions has been taking place in a progressive manner and, therefore, the previous goals have been attained). This will include a session in which a friendly (5 on 5) match will be played against a team from outside of the educational center. Such a new and different challenge will create stimuli to which youngsters are not accustomed to (cognitive dissonance).

During the “relational time,” the youngsters were made aware of how to host the visiting team. A metaphor was used in the form of “as when hosting someone at home,” including all the behaviors deemed socially acceptable, to which it was requested that the youngsters agree to abide by.

The “initial instruction” phase was used to highlight the goals that were sought with that particular match, as well as its rules. During the “training time,” and especially during the match, a guiding premise concerning self-regulating behaviors was conveyed using the metaphor “each person is his or her own referee.” The latter means that it was solicited that, should the youngsters sense they had committed an anti-sportsmanship action, they would acknowledge it at once, so that the foul could be signaled accordingly. Such behavior looks to make the youngsters aware and in-line with self-regulation and self-direction, thus facilitating the transference to life outside of training. This means that it is sought to make each individual capable of community life within a context of responsibility for one’s own choices and decisions. At the end of the match, during the “team meeting,” a congratulatory positive feedback was given to the team concerning its performance on the field. Additionally, the youngsters also shared that they felt accomplished and happy with both their individual and collective performance, which had culminated in a victory. The “reflection time” sought to encourage an exchange of ideas and a debate concerning the fundamental aspects of the session, with each youngster proceeding to perform the stretching and cooldown routine in the company of a player from the visiting team. This period included a remark that rose above the rest, as one of the youngsters approached the opposing team by saying “congrats on the match, and thanks for coming.”

### Participants and Procedures

The present study is part of an intervention for behavior modification based on a quasi-experimental design of randomized control testing. The study is part of a program for developing life skills through football in Educational Centres, with the financial backing of Portugal’s Association of Olympic Athletes, which resulted from the National Program of Sport for All, governed by the Portuguese Institute of Sport and Youth. The goal of the study is to examine the effects of different types of strategies within the program, based on the development of social and personal responsibility, so as to capacitate youngsters who have been institutionalized. The selection criteria included youngsters who had been institutionalized in educational centers in the Lisbon area, who were under educational measures, and who were between 14 and 18 years old. Of the six educational centers present in Portugal’s mainland, three of them are located in Lisbon. In pursuing this project, two of the centers were advised by the General Direction of Reinstatement and Prison Services. Consequently, the intervention took place in the Padre António Oliveira Educational Centre (in Caxias) and in the Educational Centre of Bela Vista (Lisbon, Portugal). Under these circumstances, all of the selected youngsters were male, fell within the same age range (14–18 years old) and showed similar competitive levels (Attachment 3—Demographic data). The sample was composed of 53 male youngsters (Intervention *_group_* = 27; Control *_group_* = 26). We shall now continue by describing in a detailed manner the traits of each group.

The teacher who conducted the control group sessions received TPSR-based instructor training and followed the protocol proposed by Toivonen et al. (2021). On the other hand, the teacher in the control group did not have nor did he receive any training in the TPSR model. All lessons were supervised and recorded by the first author of this study which is specialized in the TPSR model. More to the point, these recordings proved very useful in the discussion about the quality of the intervention.

#### Intervention Group

The intervention group consisted of 27 youngsters. It was subjected to an evaluation that took place in two moments, more specifically at the start and at the end of the intervention, which we designated as pre-test and post-test, respectively. In each training session, during the initial instruction, the theoretical theme chosen according to the TPSR model was presented and discussed. This meant that each session included the development of one theme, with the intervention totaling 18 sessions (for more details about levels of responsibility and behaviors to achieve consult Table 1). The theme that was chosen was always debated before, during (whenever opportunity arose), and after each training session. There was a concern with following a progressive line of action that suited the level of complexity of both the themes and the practical exercises, meaning that there was a proposal of mental exercises (e.g., focus on helping others when the situation so required) that brought to life the traits of the Hellison (2011) model.

#### Control Group

The control group, composed of 26 youngsters, continued taking part in Physical Education classes, which followed the national curriculum. This included a frequency of three weekly classes. The subjects making up the control group are of the same age range and the same gender. What distinguished the control group from the experimental group was the absence of the TPSR model from their sessions.

### Measures

To measure the levels of personal and social responsibility, we used the Portuguese version of the PSRQ by Martins et al. (2015). Originally, the PSRQ was developed and proposed by Li et al. (2008). The scale is composed by two constructs (Table 2), in which personal responsibility reflects the personal responsibilities needed in order to establish a positive learning environment (i.e., effort and self-direction), and is made up by four items (e.g., “I work hard,” “I set the goals for myself,” “I want to improve,” “I make an effort”). Social responsibility (i.e., respect for others and responsibility to care for others) also consists of 4 items (e.g., “I respect others,” “I help others,” “I encourage others,” “I am pleasant toward others”). All of the items were measured using a 6 point Likert type scale, ranging from 1 (totally disagree) to 6 (totally agree).

**Table 2:** Dimensions, description and items concerning the Portuguese version of the personal and social responsibility (Martins et al., 2015).

Dimension	Description	Items
Personal responsibility (PR)	Means accepting certain tasks as mandatory, as a result of being an individual who is a part of a community; while expecting to fulfil them properly, according to his own skill level; and acknowledging responsibility for the consequences of one’s own personal decisions.	1. I make an effort 2. I set personal goals 3. I want to improve 4. I give my best
Social responsibility (SP)	It is a multi-dimension concept that implies a sense of purpose and connectedness with others. It can be understood as a degree of compromise in the backing and support of others.	5. I respect others 6. I help others 7. I encourage others 8. I am nice toward others

The Personal and Social Responsibility Questionnaire (PSRQ) was applied before the Physical Education classes (according to the scheduling in place, and upon notifying the teachers responsible for each class and attaining their approval). Regarding the instructions, and though they were written on the questionnaires, we find it helpful to engage youngster’s to participate by reading them out loud, thus highlighting the goal of the study, the circumstance of anonymity, confidentiality, and the non-obligation to comply with filling out the questionnaire. Lastly, it was highlighted how important it was for the participants to be as honest and sincere as possible in their answers.

### Data Analysis

The data analysis was generated using the 24th version of the statistical software SPSS (SPSS 24 Inc. Chicago, IL). Table 3 showcases the 16 items that were evaluated while applying the questionnaires to the intervention group. All non-numerical data of the questionnaire were coded, namely, gender, age range, school level, sport practice, type of sport practiced, and the time intervals defined as time of sport practice.

**Table 3:** Mean, standard deviation and internal consistency of the PSRQ.

Dimensions	Items	Pre Intervention Mean (SD)	Pos intervention Mean (SD)
PR Respect	1. I respect others	4.27 (1.02)	5 (0.48)
PR Concerned with and helpful to others	2. I help others	4.27 (1.06)	4.5 (0.97)
	3. I am kind toward my colleagues	3.65 (1.17)	3.61 (1.18)
	4. I am useful to my colleagues	4.08 (1.33)	4.11 (1.0)
SR Effort	5. I make an effort	4.67 (1.21)	4.94 (0.90)
	6. I give it my best	4.44 (1.12)	5.0 (0.88)
SR Self-Direction	7. I set personal goals	4.61 (0.97)	5.0 (1.11)
	8. I want to improve	5.28 (0.74)	5.28 (0.74)
Cronbach Alfa		0.85	

The data analysis comprised several techniques, namely, the estimation of the mean and standard deviation, as well as the distribution, both absolute and relative frequencies. To verify the normality of the sample, we used the Shapiro–Wilks test. The internal consistency of the scale was verified through the calculation of the Cronbach alpha. The comparison of means and the significance test were conducted using the non-parametric tests for paired samples, namely, the Wilcoxon test for two samples concerning dichotomous nominal variables. As for the comparison of multiple means, the ANOVA one-way test was used, while being complemented with the Bonferroni post-hoc test. To test the association between the PSR constructs, the Spearman correlation coefficient was used. The level of significance used when interpreting and analyzing the data was that of *p* ≤ 0.05 for all the statistical tests.

## Results

The results of the Shapiro–Wilks tests showed the absence of normality by means of the scores varying between *W* = 0.20, *p* ≤ 0.001 and W = 0.39 *p* ≤ 0.001. Additionally, the results of the Cronbach Alfa test showed a very good internal consistency of the scale (*α* = 0.85).

As for the items to the PSR questionnaire, the results showed that the average scores displayed in the post-test were mainly greater than those recorded in the pre-test, revealing an increase of the levels of PSR, both within the personal responsibility and the social responsibility sub-scales. In the context of personal responsibility, though the variable “(PR_Self-direction) I want to improve” did not showcase any change of its average scores from the pre to the post-test, its closest score was that of 6 (*M* = 5.28; DP = 0.74). In the context of social responsibility, the variable “(SR_Being concerned with and helpful to others) I am pleasant toward my colleagues” also failed to record any positive change.

For each construct of the PSR dimension, the following intervention group variables were analyzed: Effort, Self-direction, Respect, Being concerned with and helpful to others, PR, SR, and PSR. This way, it was verified that all the variables were correlated among themselves (Table 4). Furthermore, the variable with the highest mean was “Respect,” while the one with the lowest mean was “Social responsibility” (*M* = 4.31, *SD* = 0.71).

**Table 4:** Correlation of variables of PSR.

Constructs	M	SD	1	2	3	4	5	6	7
1. Personal and social responsibility	4.63	0.63	1						
2. Social responsibility	4.31	0.71	0.92^**^						
3. Personal responsibility	4.96	0.66	0.91^**^	0.68^**^	1				
4. Effort	4.97	0.84	0.72^**^	0.45^*^	0.88^**^	1			
5. Self-direction	4.94	0.71	0.85^**^	0.74^**^	0.83^**^	0.47^*^	1		
6. Respect	5.00	0.48	0.50^**^	0.51^**^	0.39^*^	0.14	0.57^**^	1	
7. Concerned with and helpful to others	4.07	0.87	0.90^**^	0.99^**^	0.66^**^	0.46^*^	0.69^**^	0.37	1

^*^p ≤ 0.05; ^**^p ≤ 0.01.

To check for the impact of the intervention based on the personal and social responsibility of Hellison’s model, an initial analysis of the data was conducted using descriptive analysis, thus determining the number of subjects that make up the group being intervened on. We learnt that there was a global evolution of the program among the 27 subjects. There were differences in all the items, with the exception of “(SR_ Being concerned with and helpful to others) I am pleasant toward my colleagues,” in which the difference from the pre to the post-test was positive (Diff. = 0.4). These same results can be found on Table 5 for each item, before and after the intervention.

**Table 5:** Descriptive analysis of the mean and standard deviation concerning the dimension of PSR of the intervention group.

	Before		After	
Dimensions and items						
	Mean	SD	Mean	SD	Diff.	N
PSR—Personal and social responsibility	4.32	0.79	4.63	0.63	−0.31	27
PR—Personal responsibility	4.56	0.89	4.95	0.66	−0.39	27
SR—Social responsibility	4.07	0.87	4.30	0.70	−0.23	27
(SR_Respect) I respect others	4.27	1.02	5.00	0.48	−0.73	27
(SR_Being concerned with and helpful to others) I help others	4.27	1.06	4.50	.97	−0.23	27
(SR_ Being concerned with and helpful to others) I am pleasant toward my colleagues	3.65	1.17	3.61	1.18	0.04	27
(SR_ Being concerned with and helpful to others) I am useful to my colleagues	4.08	1.33	4.11	0.10	−0.03	27
(PR_Effort) I make an effort	4.67	1.21	4.94	0.90	−0.27	27
(PR_Effort) I give it my best	4.44	1.12	5.00	0.88	−0.56	27
(PR_ Self-Direction) I set personal goals	4.15	1.10	4.61	0.97	−0.46	27
(PR_ Self-Direction) I want to improve	5.00	1.11	5.28	0.72	−0.28	27

*PR, Personal Responsibility; SR, Social Responsibility; Diff., Differences; p ≤ 0.05*.

Table 6 allowed for the conclusion that, within the personal responsibility dimension, the variable “1-Effort” showcased significant differences (*t* = 0.84, *p* = 0.04), as did the variable “2. Self-direction” (*t* = 0.89, *p* = 0.08, respectively). In the social dimension, the variable “4-Respect” also showcased results deemed statistically significant (*t* = 0.65, *p* = 0.001).

**Table 6:** Multiple factor comparison tests (Lambda of Wilks), power and effect of the variables in the intervention group.

	Mean–post	
			Score	*Z*	Sig.	*η^2^_P_*	π
	Pre	Post
**1st order factor PR-intervention**							
Effort	4.56	4.97	0.84	4.93	0.04^**^	0.16	0.57
Self-direction	4.58	4.94	0.89	3.35	0.08^*^	0.11	0.42
**SR-intervention**							
Being concerned with and helpful to others	4.08	4.07	0.99	0.14	0.71	0.001	0.06
Respect	4.27	5.00	0.65	13.83	0.001^***^	0.35	0.95
**2nd order factors**							
PR_intervention	4.57	4.96	0.85	4.56	0.04^**^	0.04	0.54
SR_intervention	4.07	4.31	0.93	1.90	0.18	0.07	0.26
**3rd order factor**							
PSR_intervention	4.31	4.63	0.87	3.92	0.06^*^	0.13	0.48

PR, Personal responsibility; SR, Social responsibility; PSR, Personal and social responsibility; *p ≤ 0.1; **p ≤ 0.05; ***p ≤ 0.01.

The comparative analysis between the moments of intervention (Table 7), taking into account the several dimensions revealed that personal and social responsibility (PSR_intervention) showcased differences with scores deemed statistically significant at 90% of confidence (*t* = 0.87, *p* = 0.06). As for the personal responsibility dimension (PR_intervention), the results showcased statistically significant differences between the pre- and post-tests, at 95% of confidence (*t* = 0.85, *p = 0*.04). In the context of social responsibility (SR_Intervention), the results did not express a difference deemed statistically significant (*t* = 0.93, *p* = 0.18), despite the fact that the practical evolution of this dimension had a positive practical effect.

**Table 7:** Mean, standard deviation and differences between means, of the intervention group and the control group.

Dimensions	Control		Intervention		Differ.	Sig.
	Mean	SD	Mean	SD
(SR_Respect) I respect others	5.00	0.09	4.96	0.22	0.04	0.25
(SR_Being concerned with and helpful to others) I help others	4.81	0.21	4.50	0.19	0.31	0.10*
(SR_ Being concerned with and helpful to others) I am pleasant toward my colleagues	4.42	0.23	3.61	0.23	0.81	0.01**
(SR_ Being concerned with and helpful to others) I am useful to my colleagues	4.19	0.19	4.11	0.19	0.08	0.40
(PR_Effort) I make an effort	4.96	0.27	4.94	0.17	0.02	0.09*
(PR_Effort) I give it my best	4.92	0.26	5.00	0.17	−0.08	0.22
(PR_ Self-Direction) I set personal goals	4.65	0.25	4.61	0.19	0.04	0.18
(PR_ Self-Direction) I want to improve	5.38	0.18	5.28	0.14	0.10	0.35

SR, Social responsibility; PR, Personal responsibility¸ Differ., Differences; *p ≤ 0.1; **p ≤ 0.05.

The control group did not take part in the program based on the TPSR model. When compared with the group being intervened on, through the Wilcoxon test, statistically significant differences were found in three distinct items: “SR_(Being concerned with and helpful to others) I help others,” “SR_(Being concerned with and helpful to others) I am pleasant towards my team-mates,” “PR_(Effort) I make an effort_pos” (Table 7).

## Discussion

The intervention based on personal and social responsibility was the main pillar of this study. According to the literature, youngsters whose condition includes both risk behaviors and a high degree of emotional and social misfit can benefit from practicing team sports. According to Hellison (2011), this entails organizing sport practice in order to supply youngsters with guidelines that make them feel an obligation to assume responsibility for their own wellbeing, as well as to contribute to the wellbeing of others. The core of PSR is supported by four pillars: 1—“Putting kids first”—which suggests applying this type of programs by placing special emphasis on youngsters; 2—“Human decency”—which highlights both the evolution of youngsters while citizens, and the promotion of positive relationships with others; 3—“Holistic self-development”—in which the physical development ought to be accompanied by one’s emotional, social and cognitive development; 4—“A way of being”—which encompasses the achievement of personal wellbeing. Given these traits, as well as the positive impact that is recorded in the literature, we considered that this strategy is both viable and well supported in supplying youngsters with a meaningful experience along their individual path of evolution as responsible citizens.

The main goals of the TPSR model, also referred to as levels of responsibility, are representative of the variables that were studied (PSR, PR, SR). They can be associated with the first and second levels—“Respect” and “Effort,” respectively. The third level is associated with the variable “Self-direction” and, lastly, levels four and five with—“Help,” “Concern for others” and “Transference to outside of training,” respectively. According to Hellison (2011), the levels are categorized as follows: Beginning (levels one and two); Advanced (levels three and four); Most advanced (level five). The levels exist within a logic of progression, which entails the gradual development of the individual from the starting point of controlling one’s emotions. The relationship with others and the positive integration with the environment are the most demanding explicit goals. The social dimension is, therefore, the one that is most difficult to bring to life.

In this study, the results showed that the intervention based on the TPSR constructs had a positive impact on the intervention group. Case in point, significant differences were found in the dimensions PSR (*t* = 0.87, *p* = 0.06) and PR (*t* = 0.85; *p* = 0.04). Though no statistically significant differences were found in the variable social responsibility, the gains acquired by the youngsters in this dimension throughout the project were visible, namely, through the observation of their behavior on the field and the students’ attitudes during the final phase of the class (moment of reflection). At the start of the intervention, we were confronted with some behaviors that reflected an absence of PSR principles. For example, some statements made by the youngsters during the first sessions were:

student z—“In here there are no friends!”
student w—“This guy is full of himself!”
student k—“I set my own pace, I walk as I wish!”

By the end of the program, we were also able to record, as field notes, positive remarks that are in-line with a positive evolution of the process. These included:

1-student x: “Teacher, may I help in the data analysis at the end of the study?”
2-student y: “Never had anyone given us so much!”
3-student z: “During the weekend I shall be sending you a facebook request!”

Buying into a more self-regulated and disciplined behavior, which reflects the acceptance of new rules, became a common occurrence in the sessions. A dynamic of student/teacher and teacher/student cooperation became increasingly more evident, having witnessed a warm and positive relationship climate. The PR result that was attained was confirmed by the statistical scores on Table 6 (*t* = 0.85, *p* = 0.04), noting that this theoretical outcome became closer to the practical component.

Through these more sensitive tests (Table 6), it was possible to compare the variables of the first order, thus recording significant differences in the following: “1-Effort” (*t* = 0.84, *p* = 0.04); “2-Self-direction” (*t* = 0.89, *p* = 0.08) and “4-Respect” (*t* = 0.65, *p* = 0.001). These data support the idea that the youngsters of the Educational Centres in the Lisbon area successfully completed, after 18 sessions, the “Beginning” category (levels one and two), finishing on the transition toward level 3 “Advanced” of the TPSR model.

Over the course of the sessions individuals were stimulated to explore, so that youngsters would experience new skills and tasks. By the end of the intervention, the youngsters showcased behaviors that support the idea that there was an increase of both their motivational levels and their desire to persevere, even when the task was being challenging (“1-Effort”). One such example was that of the physical conditioning sessions, as they were quite demanding. In such sessions, we witnessed both satisfaction and good mood in accepting the task being presented. The development of these skills was also clear during the sessions that brought together rigorous drills on the technique and tactics of the sport. Though these sessions called for the significant repetition of the same task, the level of participation and commitment was never questioned.

Apart from the statistically significant results, the variable “2-Self-direction” also manifested itself in the behavior of the youngsters. When we began our intervention, the youngsters had been characterized as very reactive to failure. However, with the continued application of the program, they showcased increased levels of resistance to peer pressure. They started being able to distinguish the tasks from each other and, additionally, began playing according to their own individual purpose on the field. The performance of independent tasks constituted a strategy for acquiring skills associated with this variable. Case in point, the youngsters who showcased more aggressive and intolerant behavior, and who responded inadequately to difficult and frustrating situations, felt both more insecure and inferior to others. Consequently, they enjoyed leading, organizing and dominating. These data are in-line with the results found by Pelegrín-Muñoz et al. (2013).

We also sought to create a positive learning environment in each of the sessions, in trying to override self-destructive and disrespectful behaviors and attitudes between the youngsters. The variable “4-Respect” was visible, as behaviors that reflect it being followed were observed, which constitutes the first step toward perceived competence. When the youngsters realised they had potential (often camouflaged by colleagues who were more gifted technically and tactically), they would volunteer for the tasks being presented, meaning that some youngsters started losing the fear associated with taking part in the activity. They would also contribute with their opinions, while others began realizing that they were intervening too much. Though they did not speak a lot, since the level of pride that characterized them was exacerbated, their actions began speaking for themselves. At the same time, the meaningful respect for the adult became gradually noticeable through leadership by example, instead of imposing either force or fear (which were familiar to them). In summary, once the whole team became cohesive, teachers and staff, the respect became a constant that was ever more consistently noticeable.

Indoor football was the chosen sport for the intervention that was sought. This team game has shown to be an excellent tool for getting individuals to free themselves of their usual constraints, thus experiencing their motor skills by freely exploring and expressing themselves (Gréhaigne et al., 1997, 2005; Blomqvist et al., 2005).

Finally, as posited in the literature, a physical activity- and sport-based intervention allows for the successful implementation of social responsibility among institutionalized youngsters at risk. Therefore, the PSR model can also be a useful tool for the positive development through sport and physical activity within environments in which the youngsters have had their freedom taken away from them (Cecchini et al., 2007).

Aligned with the literature (Coulson et al., 2012), we feel that the training being supplied in the Sport Sciences courses for professionals ought to focus more explicitly on the PSR model. We believe that, by following such strategy, future professionals will be better equipped in order to be more effective and efficient teaching agents within this particular context, thus expanding the range of their professional interventions (Hemphill et al., 2013; Romar et al., 2015).

Over the brief time during which we were in contact with the youngsters, we came to understand that their talent and plans of a future faded little by little. Many of them actually had potential to play football, as well as personal traits that were quite interesting. Nevertheless, due to their family environment and/or the conditions in which they found themselves in, those qualities were being “smothered.” Surely enough, they did display behaviors that were harmful both to themselves and to society, having been punished for it (Carvalho, 2012). However, is not it up to us, as certified and worthy professionals in this field, to maximize any good quality displayed by these youngsters and, consequently, develop them as citizens? And, a sentiment that was felt while experiencing these environments as a teacher, and which will hopefully be useful to future interventions: the first impact is described as a “gaining of trust” and a constant, but healthy, “challenge” between teacher/student. From the moment that such barrier was overcome, I felt I could trust them and they could trust me. It was important to define a limit around which the youngsters felt that we were bound together, though not mixed. When the collaboration of a student was needed (recording, filling out of the questionnaires, storing the equipment), they would immediately volunteer and, though the task took place at the same time as the main activity, it did not generate instability. This type of action was experienced in both centers and also with different youngsters, meaning that this cannot be deemed as something that happened by chance or luck. Finally, the following warrants reflecting: When training sessions/classes are less successful, or the athletes/students were rude, did the coach/teacher give the best of himself from the very first contact with the youngsters? Did he show coherence and progression in all the procedures making up the class? Did he always persevere, thus creating strategies for problems to be solved?

A key aspect concerning our experience in the Educational Centres was the fact that it allowed us to contact several people and organizations. More to the point, the Educational Centres were not equipped with highly developed technicians, nor with lots of high-quality equipment. Nonetheless, upon contacting the Portuguese Association of Olympic Athletes, the Portuguese Institute of Sport and Youth, and the Nacional Plan for Ethics in Sport, it was possible to gather the solutions needed for this intervention (sports fields, goals, basketball baskets, mattresses, sports gear for the youngsters and balls). This was a noticeable outcome of this study, which might have allowed us to make a liaison that otherwise would not happen or at least it would be unlikely to happen (Darling-Hammond and Richardson, 2009).

In sum, the TPSR intervention group obtained an increase in post-test levels of personal and social responsibility, prosocial behavior, and self-efficacy due to the application of the TPSR model; compared with the control group, which used a conventional sport teaching methodology. The first remark is that the TPSR model has the potential to be adapted and implemented with flexibility in youth sport competition contexts, in order to improve personal and social responsibility, prosocial behavior, and self-efficacy. The latter is in-line with several studies Carreres-Ponsoda et al. (2021) and Martins et al. (2021). In fact, consistent with literature (e.g., Santos et al., 2020), the interest shown by the political authorities was sparked by how the development of personal and social skills transferred “out of the gym” or rather, into the everyday life of the young people at risk (Santos et al., 2020).

Another remark is that the Personal and Social Responsibility Model can be applied to institutionalized youth and is adaptable to any environment where personal and social development through sport is sought. As posited by González-Víllora et al. (2019), teaching for personal and social responsibility through cooperative learning favors its implementation, since doing so can promote outcomes in social and affective domains (e.g., psychological, social and personal development). It is important to also highlight that teachers’ engagement, training and experience are both needed and considered key features for a successful implementation (González-Víllora et al., 2019).

## Conclusion

Although the sample was small, which limits the ability to generalize the results, we underline the importance of this study for its originality. To date, there are no other studies with institutionalized young offenders. In fact, it is very difficult to access this type of institution, insofar as the government does not allow the youngsters to contact anyone other than the educational technicians or the parents. In this case, the importance of this study was recognized by the government of Portugal, in the sense that the model TPSR was deemed to be a valid tool for the personal and social development of institutionalized youngsters (Manzano-Sánchez and Valero-Valenzuela, 2019).

Finally, this study is in line with Martinek and Hellison’s (2016) demand for the future of TPSR. The authors argue that the future will require active participation and sharing of ideas among all stakeholders across governance systems and society, for this will generate new ideas that will make use of TPSR more common in its application within various scenarios (Martinek and Hellison, 2016).

## Limitations and Future Research

As any other research, this study includes some limitations. First, the number of participants is low, limiting the generalization of these results. Second, due to the end of access authorizations to educational centers, we did not conduct follow-ups, making this of interest for future studies to explore. In doing so, researchers will be able to ensure the conditions that are needed in order to measure the effectiveness of the level 5 of the TPSR program—“Outside of the gym.” Third, future research should seek the application of the model by all teachers of the educational center involved with the various groups of the same type of educational measure. This is also why these teachers should receive training on the TPSR model (Manzano-Sánchez et al. 2020). Finally, the video analysis was a method that consumed a significant amount of time, while also being difficult to conduct, since the quality of the footage was not ideal (due to the lighting conditions of the practice facilities and the players not having their name on the jerseys). The latter made it difficult to identify the subjects on some occasions, especially during the earlier stages of the study. We had, in one of the Educational Centres, only one practice field at our disposal, which made video recording and data collection more challenging during rainy days. Using paper and pen was deemed impractical, the recording camera was not waterproof, and no computers or cell phones were allowed in the premises. Despite this, the youngsters accepted to train in the rain, and the equipment was covered using umbrellas. These circumstances did, however, strengthen the bond between teacher and students, as the group came to feel that we were all united for a common purpose, regardless of the conditions. This was yet another practical, on-the-field lesson, as a problem was transformed into a life skill.

It would be interesting to continue with this sort of community program. Therefore, future studies ought to focus on populations with different traits. Doing so will allow for the analysis of the impact of this sort of intervention in socio-cultural contexts that differ from the one analyzed at this stage. Though the results were positive, little had been done thus far concerning this topic within the current Portuguese setting. Few studies have, up until now, focused on the transmission of life skills to youngsters at risk through sport. Of the few studies available, none has analyzed nor compared the results concerning the dimension of PSR and performance.

## Data Availability Statement

The raw data supporting the conclusions of this article will be made available by the authors, without undue reservation.

## Ethics Statement

Ethical review and approval was not required for the study on human participants in accordance with the local legislation and institutional requirements. Written informed consent to participate in this study was provided by the participants’ legal guardian/next of kin.

## Author Contributions

PM worked on conceptualization, data collecting, analysis, and writing. A-JG worked on the analysis and writing. ML worked on the analysis and writing. LP, as an English native speaker, worked on the translation and writing. JF conducted the classes of the program and worked on the data analysis and writing. All authors listed have made a substantial, direct, and intellectual contribution to the work and approved it for publication.

## Conflict of Interest

The authors declare that the research was conducted in the absence of any commercial or financial relationships that could be construed as a potential conflict of interest.

## Publisher’s Note

All claims expressed in this article are solely those of the authors and do not necessarily represent those of their affiliated organizations, or those of the publisher, the editors and the reviewers. Any product that may be evaluated in this article, or claim that may be made by its manufacturer, is not guaranteed or endorsed by the publisher.
